# Drug-induced cystitis caused by herbal medicine (Bofutsushosan)

**DOI:** 10.1016/j.eucr.2021.101644

**Published:** 2021-03-19

**Authors:** Kumiko Kato, Aika Matsushita, Shoji Suzuki, Hiroki Sai, Hiroki Hirabayashi, Ryohei Hattori

**Affiliations:** a Department of Female Urology, Japanese Red Cross Nagoya First Hospital, Nagoya, Japan; b Department of Urology, Japanese Red Cross Nagoya First Hospital, Nagoya, Japan

**Keywords:** Allergic cystitis, Bofutsushosan (BTS), Drug-induced cystitis, Herbal medicine, Kampo medicine, Sterile pyuria

## Abstract

Bofutsushosan (BTS), one of many traditional Japanese medicines (Kampo medicines) is attracting attention for obesity and metabolic syndrome. We report allergic cystitis caused by 8-year BTS usage in a 70-year-old female. The patient presented with micturition pain with sterile pyuria over a 3-month period. Cystoscopy showed diffuse urothelial erythema and edema. Urine cytology specimens showed increased eosinophilic cells. By discontinuing BTS, the cystitis symptoms disappeared after 4 days, and urinalysis normalized. Resuming BTS without physician approval resulted in cystitis symptoms and after cessation the symptoms rapidly subsided. This is the first English case report linking allergic cystitis to herbal medicine.

## Introduction

Traditional Japanese medicine (Kampo medicine) was derived from Chinese herbal medicine and has since developed independently. Bofutsushosan (BTS), Fangfengtongshengsan in Chinese, is a formula in Kampo medicine. It has been used for constipation in Asian countries and nowadays is attracting attention for obesity and obesity-related syndromes.^1^^,^^2^ Here, we report a case of allergic cystitis with delayed diagnosis caused by 8 years of BTS usage.

## Case report

A 70-year-old woman was referred to our department as micturition pain and lower abdominal pain did not respond to antibiotics. The patient underwent transobturator sling procedure (TOT) 10 years prior to treat stress urinary incontinence. The patient's medication history showed that she had been using BTS for 8 years to manage constipation. However, we did not feel that this was a factor in her diagnosis.

Urinalysis and urine culture showed sterile pyuria. Urine cytology showed an increase of white blood cells with no atypical cells. Maximum uroflow rate was 20.5 ml/s with 11 ml of residual urine. Bladder tuberculosis was ruled out with a negative acid-fast bacteria culture. Cystoscopy showed partial mucosal reddening without TOT mesh tape exposure. An abdominal CT-scan did not show stones, tumors or hydronephrosis, but the bladder wall was slightly thicker than usual.

**Fig. 1:**
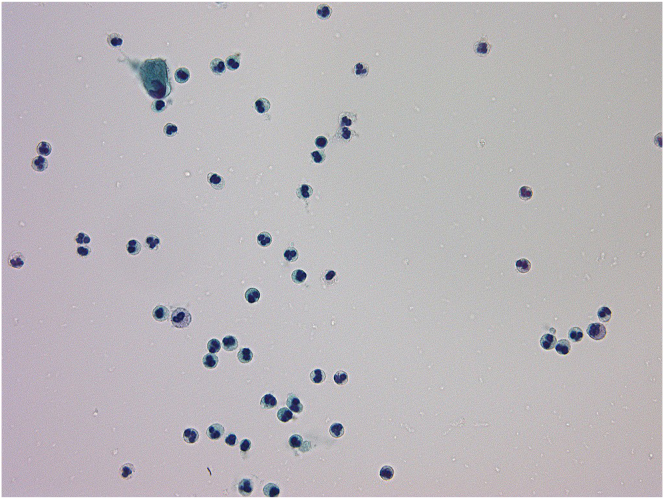
Analysis of urine cytology specimens showed increases of white blood cells including eosinophilic cells.

After 3 months of aggravated symptoms, a second cystoscopy showed diffuse white-coated mucosa with reddening and edema resembling tranilast-induced cystitis. Further literature research found 15 case reports of Kampo-induced cystitis written in Japanese.^3^ Once we suspected allergic cystitis caused by BTS, we instructed the patient to stop usage without bladder biopsy. By discontinuing BTS, the cystitis symptoms completely disappeared after 4 days, and urinalysis normalized. Analysis of former urine cytology specimens showed increases of eosinophilic cells (Fig. 1.).

Four months later, the patient resumed taking BTS without physician approval which resulted in cystitis symptoms reoccurring 3 days later. Subsequently, the patient stopped taking BTS again and the symptoms subsided within 2 days.

## Discussion

In non-bacterial cystitis, various diseases including drug-induced cystitis must be ruled out.^4^ In addition to tranilast-induced cystitis, we found 15 case reports written in Japanese on allergic cystitis caused by Japanese traditional medicine (Kampo medicine).^3^ Tsumura & Co. database showed 95 cases of Kampo-induced cystitis since 1994, 4 of which were caused by BTS (the rate of cystitis caused by BTS was estimated as 0.0001%) (personal communication). Internal Medicine, an official organ of the Japanese Society of Internal Medicine, published a review on Kampo medicine in February 2021.^5^ It summarized that most cases of interstitial pneumonia, liver injury, and allergic cystitis associated with Kampo formulas have been reported to be caused by formulas containing Scutellariae Radix (Scutellaria root, ogon). Although the detailed mechanisms are not clarified, it is generally assumed that immunoallergic reactions are involved.

The Japanese reported cases of Kampo-induced cystitis showed sterile pyuria and had similar cystoscopic findings to our case: mucosal reddening, edema, ulcer and pseudotumor. As our case underwent TOT procedure previously for stress urinary incontinence, it was necessary to rule out mesh exposure to the urinary tract. Both cystoscopy and an abdominal CT-scan showed mucosal thickening but excluded mesh exposure and stones. Japanese case reports showed that Kampo-induced cystitis could cause thickness and protrusion of the bladder wall similar to that of other drug-induced cystitis.^3^^,^^4^

The findings to support drug-induced cystitis are mainly 1) the cure of cystitis after ceasing the causative drug (removal of antigen), and 2) the pathological finding of eosinophilic cystitis.^3^ Regarding point 1, in our case, the symptoms and sterile pyuria completely disappeared after ceasing the usage of BTS. Furthermore, when the patient restarted BTS without physician approval, the symptoms recurred, and then diminished after its cessation. This unintended challenge test confirmed test-retest reliability and causal association was obvious. As for point 2, our case did not undergo bladder biopsy. Bladder biopsy can rule out bladder cancer (especially carcinoma in situ) and often shows eosinophilic infiltration in cases of allergic cystitis. However, such pathological findings are not specific. As bladder biopsy is an invasive procedure, we think that it should only be carried out if discontinuing the drug does not relieve symptoms. Urine cytology specimens showed increases of eosinophilic cells suggesting eosinophilic cystitis.

In Japan, Kampo drugs including BTS are covered by medical insurance system and physicians can prescribe them in medical institutions.^5^ BTS has also become popular as an OTC drug for obesity and metabolic syndrome. Tourists visiting Japan often buy BTS as a souvenir and it is widely available through online importers/exporters. According to the Japan Kampo Medicines Manufacturers Association (JKMA), the annual production value of BTS in 2018, approximately 31.9 billion yen (29.2 million USD), was the 11th highest of all Kampo drugs (https://www.nikkankyo.org/serv/movement/h30/all.pdf). It increased by 10% comparing with the previous year. People tend to consider herbal medicine relatively safe with few adverse effects, but it is important to be aware that herbal medicine can cause intractable cystitis which can be cured by its cessation. As it can even occur after taking Kampo drugs for long periods without symptoms (6 months–10 years), it is important to check medication history including recent and long-term usage.

## Author participations

K Kato, A Matsuyama: Conceptualization, Formal analysis, Writing.

Shoji Suzuki, Hiroki Sai: Data curation.

H Hirabayashi, R Hattori: Visualization, Supervision.

## Funding

This research did not receive any specific grant from funding agencies in the public, commercial, or not-for-profit sectors.

## Consent

Written informed consent was obtained from the patients for publication of this case report and any accompanying images.

## Declaration of competing interest

None.
